# Deficient Explicit Access to Phonological Representations Explains Phonological Fluency Difficulties in Greek Children With Dyslexia and/or Developmental Language Disorder

**DOI:** 10.3389/fpsyg.2019.00638

**Published:** 2019-04-11

**Authors:** Maria Mengisidou, Chloë R. Marshall

**Affiliations:** Department of Psychology and Human Development, UCL Institute of Education, University College London, London, United Kingdom

**Keywords:** dyslexia, developmental language disorder, phonological fluency, phonological deficit, phonological representations, deficient access, design fluency, Greek

## Abstract

It is well-established that children with dyslexia and/or Developmental Language Disorder (hereafter children with DDLD) perform poorly on phonological tasks compared to typically developing (TD) children. However, there has been some debate as to whether their phonological deficit arises directly from an impairment in phonological representations, or instead from deficient access to (intact) phonological representations. This study tested the Degraded Phonological Representations Hypothesis and the Deficient Phonological Access Hypothesis using a task that is not often used with children with DDLD, namely phonological fluency. Both hypotheses predict that children with DDLD will retrieve fewer items than their TD peers in the phonological fluency task. However, while the Degraded Phonological Representations Hypothesis predicts smaller clusters of phonologically related items in children with DDLD, the Deficient Phonological Access Hypothesis predicts that the two groups will not differ in cluster size. How phonological fluency performance related to children’s language, literacy, and phonological skills was investigated. Further, the specificity of a phonological fluency deficit in children with DDLD was tested using a nonverbal (design) fluency task. Sixty-six children with DDLD aged 7–12 years and 83 TD children aged 6–12 years, all monolingual Greek speakers, were tested on three phonological fluency categories, on nonverbal IQ, language, literacy, and phonological tasks, and on a design fluency task. The DDLD group produced significantly fewer correct responses and fewer switches compared to the TD group, but the two groups showed similar clustering and average cluster size. After controlling for age, children’s language, literacy, and phonological skills significantly predicted the number of correct responses produced. The two groups did not differ significantly on the number of unique designs generated in the design fluency task. Furthermore, children with DDLD showed poorer phonological fluency performance relative to their TD peers even after design fluency performance was controlled, demonstrating the specificity of their phonological fluency deficit. This study adds to the theoretical debate on the locus of the phonological deficit in dyslexia and DLD. The findings support the hypothesis that the phonological deficit in dyslexia and DLD lies in deficient explicit access to intact phonological representations.

## Introduction

Dyslexia and Developmental Language Disorder^1^ (DDLD) are two neurodevelopmental disorders which affect, respectively, the typical development of literacy and oral language skills. It is well-established that children with dyslexia and DLD show poor performance on three main dimensions that rely on the efficient functioning of the phonological system, namely on tasks assessing phonological awareness, phonological short-term memory, and rapid automatic naming skills (see for dyslexia: Wagner and Torgesen, 1987; see for DLD: Ramus et al., 2013). Phonological representations (referring to the abstracted way that speech sounds of a particular language are represented in the brain) are involved in the successful completion of all three tasks. Specifically, the tasks require, respectively, the manipulation of phonological representations (such as deletion tasks), the retention of verbal material in short-term memory (such as nonword repetition tasks), and quick and efficient access to phonological representations (such as rapid automatic naming tasks) (e.g., Snowling, 2000). Phoneme deletion, nonword repetition, and rapid automatic naming tasks have been reported to account for significant amounts of variance in reading and spelling performance across orthographies, as evidenced by large-scale cross-linguistic studies in typically developing (TD) children (Ziegler et al., 2010; Moll et al., 2014).

Why is it that children with dyslexia and DLD show poor performance in phonological tasks and how is poor phonological ability linked to reading difficulties? Phonemes are distinct cognitive categories imposed by our phonological system upon a gradient acoustic space (Ladefoged, 2001). For example, in the case of the words “bat” and “pat,” voice onset time is a gradient cue signaling the difference between voiced /b/ and voiceless /p/ in English. In order for spoken word recognition (e.g., the recognition of the word “bat”) to proceed successfully, phonological representations must be robust (i.e., all /b/ sounds must be assigned to the same phoneme category) and distinct (i.e., /b/ sounds must be distinguished from /p/ sounds). However, while phonetic forms can be identified at a level of perception, recognition of phonological units (e.g., phonemes) involves additional cognitive processes such as categorization (Ladefoged, 2001). It follows that phonological representations are a way of storing the sound sequences that make up words in an abstracted form. Moreover, as the initial stages of reading development are characterized by learning how graphemes (i.e., letters and group of letters) map onto their corresponding sounds, it is not surprising that the consensus view for many years has been that dyslexia is the behavioral outcome of an underlying phonological deficit. This view has received substantial empirical support from a range of experimental studies (e.g., Grigorenko, 2001; Ramus et al., 2003; Vellutino et al., 2004; Saksida et al., 2016).

The language examined in the current study is Greek. Greek has a shallow orthography, which means that it is characterized by consistent grapheme-to-phoneme mappings (Seymour et al., 2003), estimated to be 95% consistent for reading and 80% consistent for spelling (Protopapas and Vlahou, 2009). Considering this high level of orthographic consistency, it is not surprising that reading difficulties are evident primarily in poor reading fluency rather than poor reading accuracy (Nikolopoulos et al., 2003). Poor reading fluency in turn is associated with poor performance on phonological awareness and rapid automatic naming tasks (Nikolopoulos et al., 2006; Protopapas et al., 2013a,b). Having said that, reading accuracy difficulties are evident in children with dyslexia even in Grade 7 (Protopapas and Skaloumbakas, 2007; Protopapas et al., 2008, 2012). With respect to phonological difficulties, children with dyslexia and DLD have been reported to show phonological deficits in tasks measuring phonological awareness, phonological short-term memory, and rapid automatic naming skills (e.g., Talli et al., 2016; Diamanti et al., 2018; Spanoudis et al., 2018). There is also evidence that relatively easy tasks for assessing phonological awareness, such as phoneme segmentation and phoneme deletion tasks, show ceiling effects by the end of Grade 1, and are not therefore able to reveal children’s phonological difficulties (Papadopoulos et al., 2009, 2012). However, more demanding phoneme deletion tasks, when stimuli comprise polysyllabic nonwords with consonant clusters, can reveal group differences in 3rd and 4th Graders (Protopapas et al., 2008), and in children with dyslexia through secondary education (Protopapas and Skaloumbakas, 2007; Anastasiou and Protopapas, 2015). With respect to phonological skills in DLD, children with DLD aged 8–12 years are reported to show poorer phonological short-term, working and long-term memory skills relative to their TD peers (Spanoudis and Natsopoulos, 2011). Further research is, however, needed to investigate the locus of the phonological deficit in Greek children with dyslexia and DLD. In the light of evidence that the manifestation of the phonological deficit in dyslexia is moderated by orthographic consistency (e.g., Wimmer, 1996; Frith, 1999; Landerl and Wimmer, 2000; Landerl et al., 2013), the objective of this study is to investigate the locus of the phonological deficit in children with dyslexia and DLD using a language whose orthography is consistent.

In order to investigate the locus of the phonological deficit in children with DDLD, the two prominent phonological hypotheses of dyslexia and DLD are considered. The leading view on dyslexia for many years has been that phonological representations are degraded (i.e., less robust and distinct), and that this primary representational deficit impacts upon higher-level phonological processing, and ultimately, upon reading development. This view is called the Degraded Phonological Representations Hypothesis (e.g., Goswami, 2000; Ziegler and Goswami, 2005; Leong et al., 2011). The concept of degraded phonological representations implies that during the course of development, children with dyslexia have experienced difficulties in establishing representations of phonological units that are adequately robust and distinct for the recognition and production of words.

However, in an influential review of the dyslexia literature in adults, Ramus and Szenkovits (2008) argued that the phonological deficit is evident only under certain task demands, namely tasks requiring explicit manipulation of speech sounds, loading phonological short-term memory, or requiring speeded access to phonological representations. The researchers instead proposed the Deficient Phonological Access Hypothesis, arguing that phonological representations of people with dyslexia are intact, but hard to access because of the involvement of the aforementioned processes, which are required to explicitly access phonological representations, processes which are deficient in dyslexia. This hypothesis has since been supported by a number of empirical studies (e.g., Soroli et al., 2010; Boets et al., 2013; Dickie et al., 2013; Ramus et al., 2013; Szenkovits et al., 2016). This hypothesis reflects a central distinction in the literature between explicit and implicit access to phonological representations: for the latter, processing demands are minimized, and it is only by using phonological tasks with minimal processing demands that the quality of phonological representations can themselves be assessed (Ramus et al., 2013).

The two phonological hypotheses of dyslexia presented also apply to DLD, given that many children with DLD have phonological difficulties linked to reading difficulties similar to those seen in children diagnosed with dyslexia (e.g., Kamhi and Catts, 1986; Catts, 1993; Bishop et al., 2009; Hulme and Snowling, 2009). From the review so far, it is evident that in the dyslexia literature, most of the studies supporting a phonological access deficit have been conducted in adults. Adopting a developmental perspective, however, allows us to test what is perhaps the most valid criticism of the Deficient Phonological Access Hypothesis: the possibility that adults with dyslexia have degraded phonological representations in childhood, but these representations have recovered by adulthood (e.g., Goswami, 2003). Overall, it is not yet clear whether the phonological deficit in dyslexia and DLD originates from degraded phonological representations themselves, or whether the phonological representations are intact but access to them is problematic whenever task demands are high. Our contribution to this debate is to test the two hypotheses using just one task – phonological fluency – which requires both explicit and implicit access to phonological representations.

Phonological fluency tasks are lexical–retrieval tasks requiring children to produce as many words as they can which begin with particular letters, usually in a 60-s test period. Word productivity, however, declines through the test period, and especially after the first 15 s have elapsed (e.g., Henry et al., 2015), suggesting that retrieval becomes harder during the course of the test period. Retrieving words beginning with particular letters would suggest that one has representations of those words in which an initial phoneme is distinct, or segmentable, from the rest of the word form (Nash and Snowling, 2008). This suggests that word productivity in phonological fluency tasks is a measure of children’s conscious, or explicit, access to phonological representations.

Further, in phonological fluency tasks, responses are often produced in clusters of phonologically related items (Troyer et al., 1997). For example, “flag-flower” is a cluster since the two words share the initial two phonemes (“fl”). We argue that phonological clustering provides a more implicit measure of the quality of children’s phonological representations on the basis that phonological similarity in successive produced responses might aid lexical retrieval. In the example given above, the retrieval of “flag” might facilitate the retrieval of “flower” because in the two words phonological representations partly overlap. Given the limited time of the test period, once lexical retrieval within a cluster slows down, individuals tend to switch to another cluster (e.g., from “flag-flower” to “free-friend”). Both clustering and switching strategies show a strong positive correlation with the number of correct items retrieved in phonological fluency tasks (e.g., Kosmidis et al., 2004). Overall, successful performance on phonological fluency tasks requires the search of the mental lexicon for words on the basis of their phonology. Importantly, the phonological fluency task measures two different aspects of access to phonological representations, namely explicit access to phonological representations, as evidenced by the number of correct responses retrieved, and implicit access to phonological representations, as evidenced by the size of clusters produced.

Mixed findings have been reported with respect to what drives children’s productivity in phonological fluency tasks, with studies reporting that productivity is associated with the number of switches and of clusters but not with average cluster size (e.g., Henry et al., 2012, 2015), and other studies reporting that productivity is predicted by cluster size in addition to the number of switches and of clusters (e.g., Arán-Filippetti and Allegri, 2011). With respect to phonological fluency performance in dyslexia, studies have reported consistent findings showing that children with dyslexia retrieve significantly fewer items than TD children (e.g., Brosnan et al., 2002; Plaza et al., 2002; Landerl et al., 2009; Varvara et al., 2014; Moura et al., 2015). Studies have also consistently reported significantly lower phonological fluency performance for English children with DLD compared to TD children (Weckerly et al., 2001; Henry et al., 2012, 2015). To our knowledge, the only published study which has investigated phonological clustering and switching patterns in children with dyslexia reported that Polish-speaking adolescents with dyslexia aged 16–18 years did not differ on the number of clusters, number of switches, or size of clusters compared to adolescents without dyslexia (Mielnik et al., 2015). With respect to DLD, Weckerly et al. (2001) found that children with DLD aged 8–12 years produced significantly fewer clusters and switches compared to TD children, but the two groups did not differ on cluster size. Henry et al. (2015) also found that children with DLD produced fewer switches, in addition to a marginally smaller number of items per cluster than TD children. We thereby argue that there is only limited evidence originating from child samples of dyslexia and DLD reporting patterns of lexical retrieval in phonological fluency tasks in languages other than English. The current study aims to fill this gap in the developmental literature by testing phonological fluency performance in Greek children with dyslexia and/or DLD. Further, evidence showing whether children’s language, literacy, and phonological skills predict phonological fluency performance in children with DDLD is scant. Given that there are still age-related improvements in phonological fluency until mid-adolescence (Hurks et al., 2010), phonological fluency tasks should be sufficiently sensitive to differentiate among primary school-aged children.

It is assumed that TD children have age-appropriate explicit and implicit access to phonological representations. In this context, the more robust and distinct children’s phonological representations are, the easier it will be for them to retrieve – by explicit access – items belonging to a phonological category. In the context of typical phonological representations, the easier it will also be for them to produce – by implicit access – a phonological cluster, such as “**st**ar-**st**are-**st**reet-**st**rong,” since all four items share the initial two phonemes, and therefore the greater the number of items produced belonging to that subcategory (i.e., the greater the cluster size) will be. Turning now to the predictions of the study, both hypotheses predict that children with dyslexia and DLD will produce fewer items compared to TD children. The pattern of retrieval predicted by the two hypotheses is, however, different. According to the Degraded Phonological Representations Hypothesis, the average size of clusters in children with DDLD will be smaller relative to TD children. This is because if phonological representations are less robust and distinct, phonological similarity is less well represented and therefore exploited less readily by children with DDLD; they will have difficulty in retrieving words in clusters, which will result in the production of smaller clusters, and fewer items overall. In contrast, the Deficient Phonological Access Hypothesis predicts that even though a phonological access deficit is evident in children with DDLD, meaning that fewer items are produced overall, cluster size should not differ between the two groups because phonological similarity is equally well represented in children with DDLD and TD children. In sum, cluster size is considered to be an implicit, and therefore more direct, measure of the quality of phonological representations, and the two hypotheses make different predictions with respect to it.

Another issue emerging from the two hypotheses is the specificity of the phonological fluency deficit in dyslexia and DLD. Protopapas (2014) argues that to establish the viability of any phonological hypothesis, one has to ensure that statistically poorer performance on tasks requiring phonological processing is accompanied by normal performance on similarly structured tasks that do not involve phonological processing. The design fluency task used in this study measures visuospatial executive skills by assessing a child’s ability to generate nonsense designs under time constraints and restricted design conditions. It is therefore a similarly structured task to phonological fluency tasks without requiring, however, phonological representations and phonological processing skills. Both the Degraded Phonological Representations Hypothesis and the Deficient Phonological Access Hypothesis predict that children with DDLD will generate a similar number of unique designs in the design fluency task compared to TD children. This is because both hypotheses advocate a “modular” deficit within the language system which affects the phonological domain, while the nonverbal domain is unaffected. However, given empirical evidence that children with dyslexia and DLD demonstrate deficits beyond the phonological system (e.g., Henry et al., 2012; Gooch et al., 2014; Varvara et al., 2014; Henry and Botting, 2017), for the purpose of this study, further investigation in the nonverbal domain is needed. Moreover, as Messer and Dockrell (2006) have argued in the context of children with word-finding difficulties, lexical–retrieval difficulties can potentially be caused by impairments in processing speed, among other proposed causes. It is hypothesized that if there is a slower processing speed in children with DDLD accounting for lower phonological fluency performance, lower design fluency performance would be also found; however, if only phonological processing difficulties were to underlie poorer phonological fluency performance in children with DDLD, the two groups would show similar design fluency performance. Further, in order to test the specificity of the phonological fluency deficit in children with DDLD, design fluency performance will be used as a covariate in the analysis investigating group differences in phonological fluency. Existing research on design fluency in children with dyslexia is limited, and inconsistent findings have been reported, with one study reporting that the dyslexia group generated significantly fewer unique designs than the TD group (Griffiths, 1991), and another study reporting no group difference (Reiter et al., 2005). To our knowledge, only one published study used design fluency in children with DLD and showed that the DLD group generated significantly fewer unique designs compared to the TD group (Henry et al., 2012). There are no design fluency data originating from Greek children with dyslexia and DLD.

In the current study, the association between language, literacy, and phonological skills and productivity in the fluency task is also considered. Previous studies have shown that greater productivity in phonological fluency tasks is associated with better performance on language measures, as reported by Henry et al. (2015) who assessed English TD children and children with DLD and by Luo et al. (2010) who assessed monolingual and bilingual English adults. Aside from the role of language ability, literacy skills have also been found to play a role in phonological fluency performance. For example, indirect evidence for the effect of literacy skills on phonological fluency performance originates from the study of Riva et al. (2000) who tested children aged 5–11 years. An important finding of their study was that phonological fluency performance increased linearly from first Grade to fifth Grade, with the most significant increase observed between first and second Graders. The authors argued that this is because formal teaching begins at that time, and children begin to develop awareness of the components of language, including phonology. Riva et al. (2000) proposed therefore an association between the development of the ability to organize words and to retrieve them according to phonological categories and reading skills. The current study will investigate for the first time whether language, literacy, and phonological skills predict word productivity in phonological fluency categories in Greek TD children and children with DDLD. Considering that children with DDLD show inferior language, literacy, and phonological skills relative to their TD peers, it is predicted that productivity in phonological fluency categories will be partly accounted for by these skills.

The study addressed the following research questions about phonological fluency in Greek-speaking children with DDLD:

• Where does the phonological deficit in children with DDLD lie? Is poorer phonological fluency performance in children with DDLD better explained by degraded phonological representations or by deficient explicit access to (intact) phonological representations?
• Do cluster number and/or cluster size drive productivity in phonological fluency tasks in TD children and children with DDLD?
• Does phonological fluency performance relate to children’s language, literacy, and phonological skills?
• How specific is the phonological fluency deficit in children with DDLD: Does it extend to a nonverbal task (design fluency)?

## Materials and Methods

### Participants

Sixty-six children with dyslexia and/or DLD (43 males) and 83 TD children (35 males), who were all monolingual Greek speakers, participated in the study. Children with dyslexia and/or DLD were selected on the basis that they had received a diagnosis because of persistent and specific reading or language problems. Thirty children with dyslexia and/or DLD had co-existing difficulties accompanying the diagnosis of persistent and specific reading or language problems, such as attention-deficit/hyperactivity disorder, developmental disorder of motor skills, articulation disorder, specific disorder in speech fluency, or dysgraphia. In line with the CATALISE consortium (Bishop et al., 2017), children with additional disorders were not excluded from the study given that additional disorders are considered as descriptors of a child’s profile. Further, five children with a lower nonverbal ability (i.e., a standard score equal to 75) in the nonverbal IQ task were also not excluded from the study, following Norbury et al.’s (2016) population study which reported that children with a lower nonverbal ability (i.e., a standard score between 70 and 85) did not differ significantly in their language profile from children with an average nonverbal ability (i.e., a standard score > 85). TD children who achieved a percentile score of 10 or lower on a standard text-reading fluency measure, or substantial difficulties with the language and literacy tasks, were excluded from the study. None of the children included in the study had a current or prior history of hearing or visual deficit, neurological disease, or medication for any neurological, psychiatric, or behavioral disorder. None scored lower than 80 on the nonverbal IQ task.

Traditionally, dyslexia and DLD are viewed as separate disorders. In this study, however, the children with dyslexia and DLD were combined in one group, the DDLD group. In fact, literacy difficulties are very common in children with DLD (e.g., Conti-Ramsden et al., 2001), and it is the case that approximately 50% of children who fit the criteria for dyslexia also fit the criteria for DLD, and vice versa (e.g., Messaoud-Galusi and Marshall, 2010; Spanoudis et al., 2018). However, there are currently no gold standard assessments for diagnosing dyslexia and DLD with adequate psychometric properties, namely, valid and reliable assessments with diagnostic or prognostic value. In this context, Dockrell and Marshall (2015) argue that screening measures to date do not meet psychometric properties to identify language problems, and also that the interpretation of language assessments is challenged by a range of factors, including socioeconomic status, multilingualism, hearing impairment, and even the characteristics of the assessment. The last of these factors is particularly relevant to the present study. In Greece, dyslexia is typically diagnosed on the basis of nonstandardized measures of reading and spelling ability (Anastasiou and Polychronopoulou, 2009), and the same is also the case for DLD. This raises the issue of how accurately children with dyslexia, children with DLD, and children with dyslexia plus DLD can be differentiated; this might not be as easy as in studies of English-speaking children (e.g., Catts et al., 2005; Ramus et al., 2013). Further, previous research in Greek has explored the overlap between dyslexia and DLD, and reported that dyslexia and DLD show common deficits in tasks measuring reading skills and reading-related phonological skills (Talli et al., 2016; Spanoudis et al., 2018), even though they do not completely overlap.

In the light of this evidence, a PCA with rotation (oblique) within the language and literacy skills of the children with dyslexia and/or DLD was carried out in order for us to determine whether there were separate loadings onto different components that might justify grouping the children with dyslexia and DLD separately. The dataset was suitable for the PCA: Kaiser–Meyer–Olkin Measure of Sampling Adequacy value was 0.787, meeting Kaiser’s (1974) criterion for this value, Bartlett’s Test of Sphericity value was significant (*p* < 0.001; Bartlett, 1954), and most of the intercorrelations observed among all seven measures of interest had a value of 0.30 and above. The PCA revealed that five language tasks (WISC similarities, WISC vocabulary, syntax comprehension, sentence repetition, and receptive vocabulary) and two literacy tasks (l’Alouette and spelling-to-dictation) used in the overall sample to profile children with dyslexia and/or DLD loaded onto component 1. Table 1 presents each task’s contribution to components 1 and 2, which is expressed by its loading value. WISC vocabulary, receptive vocabulary, and WISC similarities had the highest loadings onto the first component, while l’Alouette and sentence repetition had the lowest loadings onto this component.

**Table 1:** The loadings onto components 1 and 2 for each task generated by the PCA (with oblique rotation) in the DDLD group.

Tasks	Component 1	Component 2
WISC vocabulary	0.83	0.10
Receptive vocabulary	0.78	0.13
WISC similarities	0.80	-0.02
Spelling-to-dictation	0.70	-0.52
Syntax comprehension	0.68	0.48
L’Alouette	0.60	-0.62
Sentence repetition	0.41	0.57

Components 1 and 2 had an eigenvalue larger than 1, meeting Kaiser’s (1974) criterion. The first component had, however, by far the largest eigenvalue of all seven components generated by the PCA (3.4). The second component had an eigenvalue of 1.2 and accounted for 18% of the variance in all measures, while the remaining components had an eigenvalue lower than 1, and as such, they were not considered further. Even though components 1 and 2 had an eigenvalue larger than 1, a one-factor solution was selected. This selection was based on the scree plot generated by the PCA illustrating a clear split between component 1 and the remaining components. The PCA was therefore repeated, and a one-factor solution was selected. This analysis revealed that component 1 had an eigenvalue of 3.4 and explained 49.68% of the variance in all seven measures. If the first component had loaded essentially on language variables and the second component on literacy variables (or the other way around), then this would have been strong evidence that language and literacy variables were two distinct sources of variance in this dataset. If this had been the case, it would have been a good reason to group the children with dyslexia and DLD separately. However, the PCA revealed that language and literacy variables did not load on different components, which suggests that it is appropriate to combine the children with dyslexia and/or DLD into a single DDLD group.

This DDLD group had a mean age (*SD*, range) of 9.51 (1.46, 7;04–12;02) years and the TD group had a mean age of 8.37 (1.77, 6;03–12;04) years. The DDLD group was significantly older than the TD group, *t*(147) = -4.30, *p* < 0.001. On the Greek standardization of the nonverbal IQ task (Sideridis et al., 2015) of the Raven’s Colored Progressive Matrices (CPM; Raven, 2008), the mean standard score of the DDLD group was 96.74 (*SD* = 15.12) and of the TD group was 104.75 (12.94). The TD group significantly outperformed the DDLD group, *t*(147) = 3.48, *p* = 0.001, as has been found in previous studies of children with literacy and language disorders (e.g., Ramus et al., 2013). Nonverbal IQ was not statistically controlled in the analyses, however, following Dennis et al. (2009) who argued that using IQ scores as a covariate is misguided and unjustified in cognitive studies with children with neurodevelopmental disorders.

In order to better appreciate the DDLD group’s overall performance on language, literacy, and phonological tasks, analyses of covariance (ANCOVA) were carried out, with the score of each task as a dependent variable, group as a fixed factor, and age in months as a covariate variable. Since the two groups were not matched for age, and phonological fluency performance was related to age in each group (see the section “Results”), age was controlled in the analyses, and estimated marginal means and estimated standard error are presented. These tasks are described later, but the data are presented here in order to provide information about the language, literacy, and phonological profile of the DDLD group compared to the TD group. Table 2 shows that the TD group significantly outperformed the DDLD group in all language tasks, in all literacy tasks except for the syllable reading task, and in all phonological tasks except for the phoneme deletion task with CVC items (C: consonant; V: vowel).

**Table 2:** Groups’ performance and group differences on language, literacy, and phonological tasks.

	DDLD group		TD group					
Tasks	*e. m.* mean	*e. SE*	*e. m.* mean	*e. SE*	*n*	*F*	*p*	*ηp*^2^
Language skills								
Verbal comprehension: WISC similarities	8.16	0.42	11.13	0.37	149	26.23***	<0.001	0.152
Verbal comprehension: WISC vocabulary	14.79	0.59	21.61	0.52	149	69.91***	<0.001	0.324
Syntax comprehension: DVIQ test	12.71	0.26	13.80	0.23	149	8.37**	0.004	0.054
Sentence repetition: DVIQ test	23.44	0.48	27.09	0.43	149	29.93***	<0.001	0.170
Receptive vocabulary: PPVT-R	108.60	1.68	121.41	1.48	149	30.79***	<0.001	0.174
Literacy skills								
Text-reading fluency: L’Alouette	105.51	5.26	177.60	4.66	149	99.62***	<0.001	0.406
Text-reading fluency: reading test alpha	62.57	3.14	92.58	3.47	102	40.86***	<0.001	0.292
Reading accuracy: reading test alpha	95.07	1.36	105.06	1.50	102	24.11***	<0.001	0.196
Syllable reading: test of DIRD	20.00	1.07	22.45	0.49	47	3.82	0.057	0.080
Nonword reading: test of DIRD	16.97	1.28	21.33	0.59	47	8.40**	0.006	0.160
Spelling ability: Spelling-to-dictation	14.96	0.91	28.68	0.81	149	118.74***	<0.001	0.449
Phonological skills								
Phoneme deletion of CVCVCV items: EVALEC	6.51	0.26	8.33	0.23	149	25.51***	<0.001	0.143
Phoneme deletion of CVC items: EVALEC	10.93	0.17	11.03	0.15	149	0.17	0.675	0.001
Phoneme deletion of CCV items: EVALEC	8.79	0.28	10.57	0.25	149	20.12***	<0.001	0.130
Nonword repetition: EVALEC	13.60	0.49	18.14	0.42	149	45.48***	<0.001	0.241
Rapid automatic naming: PhAB	151.69	4.34	109.87	3.75	149	49.99***	<0.001	0.259

*WISC, Wechsler Intelligence Scale for Children; DVIQ test, Diagnostic Verbal Intelligence Test; PPVT-R, Peabody Picture Vocabulary Test-Revised; Test of DIRD, Test of Detection and Investigation of Reading Difficulties; EVALEC, Evaluation de la Lecture; PhAB, Phonological Assessment Battery; ^∗∗^*p* < 0.01, ^∗∗∗^*p* < 0.001; *ηp*^*2*^, partial eta-squared, respectively, 0.01, 0.06, and 0.14 small, medium, and large effect size.*

### Procedure

The study obtained ethical approval from the Departmental Research Ethics Committee of UCL Institute of Education’s Department of Psychology and Human Development, and from the Hellenic Ministry of Education, Research, and Religious Affairs. Parents were asked to sign a written informed consent on behalf of the children in accordance with the Declaration of Helsinki. All the children were assessed individually by the first author, a native Greek speaker, in one session lasting approximately 90 min. The children were tested in a school classroom, or in the referral center where they were receiving speech and language therapy. Audacity for Windows 7 was used to record responses for later transcription.

### Materials

A wide battery of language, literacy, and phonological tasks was used to profile the DDLD group. All the children were assessed with tasks drawing upon a range of language processing skills, namely receptive vocabulary, verbal comprehension, syntax comprehension, and sentence repetition. In the Greek shallow orthography, reading accuracy and reading fluency are sensitive measures that can reveal reading disorders (Talli et al., 2016; Diamanti et al., 2018). Spelling performance is another sensitive index of reading difficulty in the Greek orthography (Porpodas et al., 1999; Protopapas and Skaloumbakas, 2007). Two literacy tasks were used with all the children: l’Alouette and spelling-to-dictation. In addition to these two tasks, first and second Graders were also assessed with syllable and nonword reading tasks, and third to sixth Graders were also assessed with reading accuracy and text-reading fluency tasks, tasks for which normative data are available covering the age range of this study. Further, all the children were assessed with phoneme deletion, nonword repetition, and rapid automatic naming tasks measuring reading-related phonological skills, tasks in which the typical phonological deficit in children with dyslexia and DLD becomes evident (e.g., Ramus et al., 2013; Talli et al., 2016).

### Fluency Skills

#### Phonological Fluency

The phonological categories “chi,” “sigma,” and “alpha” of the Greek alphabet were used, in that order. Children were instructed to produce as many different words belonging to the target category as possible, allowing 60 s for each category. No examples were given, but the letter “tau” was used as a practice category. The number of correct responses retrieved for the three phonological categories was combined to create a composite phonological fluency score.

#### Design Fluency

The NEuroPSYchological Assessment (NEPSY; Korkman et al., 1998) design fluency subtask contains two booklets of 35 five-dot designs each. Four designs were given as practice trials. Children were given 60 s for each page to create as many different designs as fast as they can by connecting two or more dots in each square. The task measures visuospatial cognitive fluency and performance on the task is expressed as the number of unique designs in both booklets (maximum = 70).

### Language Skills

#### Verbal Comprehension

Children’s verbal comprehension skills were assessed with the similarities and vocabulary subtasks of the Greek standardization (Georgas et al., 1997) of the Wechsler Intelligence Scale for Children (WISC-III; Wechsler, 1991). For the similarities subtask, children had to identify how two words are alike (maximum score = 33). Responses scored one or zero points for the first five questions, and two, one, or zero points for the remaining questions. For the vocabulary subtask, children were asked to define words (maximum score = 60). Responses scored two, one, or zero points. In both subtasks, the difference in scores reflected the quality (accuracy and detail) of the response given.

#### Syntax Comprehension and Sentence Repetition

Syntax comprehension and sentence repetition subtasks of the Diagnostic Verbal Intelligence (DVIQ) Test (Stavrakaki and Tsimpli, 2000) were used. For syntax comprehension, children were presented orally with a sentence and had to choose the picture that best depicted the meaning of the sentence. The number of correctly chosen pictures was computed (maximum = 17). For sentence repetition, children were asked to repeat 10 sentences as accurately as possible, and the child’s maximum score was equal to 30. Responses could score three, two, one, or zero points depending on the accuracy of the response.

#### Peabody Picture Vocabulary Test-Revised (PPVT-R)

Children’s receptive vocabulary was assessed with the Greek non-standardized version (Simos et al., 2011) of the PPVT-R (Dunn and Dunn, 1981). Children were provided orally with a word and were instructed to decide which of the pictures provided best represented its meaning. The child’s score was the number of correctly selected pictures (maximum = 173).

### Literacy Skills

#### L’Alouette

L’Alouette task (Lefavrais, 1967) adapted into Greek (Talli et al., 2016) was used to assess text-reading fluency of a text bearing no meaning. The number of words read correctly within 3 min was recorded for each child (maximum = 271).

#### Reading Accuracy and Text-Reading Fluency

Reading accuracy and text-reading fluency in third to sixth Graders were assessed with the Reading Test Alpha (Panteliadou and Antoniou, 2007). The reading accuracy score was the number of words and nonwords read correctly (maximum = 77), alongside the number of words and nonwords identified as such in the lexical decision subtask (maximum = 36). The reading fluency score was the number of words read correctly within 60 s (maximum = 279).

#### Syllable and Nonword Reading

Syllable and nonword reading in first and second Graders were assessed with the Test of Detection and Investigation of Reading Difficulties (Porpodas, 2007). For each subtask, the number of syllables and nonwords read correctly was computed (maximum = 24, for each subtask).

#### Spelling-to-Dictation

The spelling-to-dictation task (Sideridis et al., 2008) consisted of 60 words. The experimenter read the word aloud, then read the word aloud in the context of a sentence, and then read the word aloud for a final time. Each correctly spelled word scored one point, and after six spelling errors in a row, the experimenter discontinued the task.

### Phonological Skills

#### Phoneme Deletion

Three phoneme deletion tasks of the computerized battery Evaluation de la Lecture (EVALEC; Sprenger-Charolles et al., 2005) adapted into Greek by Talli et al. (2016) were used to assess children’s phonological awareness skills. Phoneme deletion tasks of CVC, CCV, and CVCVCV items were used. Children had to produce the nonword without the initial consonant or consonant cluster. Prior to testing, children were asked to delete the first sound of three nonwords for each task used as practice trials. A child’s score was the total number of correct responses in each task (respectively, maximum = 12, 12, and 10).

#### Nonword Repetition

Children were instructed to repeat nonwords varying in syllable length (from three to six syllables) of the EVALEC’s nonword repetition task (Sprenger-Charolles et al., 2005) which has been adapted into Greek (Talli et al., 2016). Prior to testing, children were asked to repeat three nonwords as practice trials. The child’s score was the number of nonwords repeated correctly (maximum = 24).

#### Rapid Automatic Naming

The picture naming subtask of the Phonological Assessment Battery (PhAB; Frederickson et al., 1997) was used to assess rapid automatic naming. The task contains two cards of five pictures repeated 10 times on each card. Prior to testing, all five pictures were named by the experimenter to ensure that the children knew pictures’ names. Children were instructed to name the pictures as fast as possible. The average naming time (in seconds) was the child’s score.

#### Coding of Phonological Fluency Responses

Responses were coded as correct, scoring one point each, or incorrect. Correct responses were considered words beginning with the target letter or with a letter having the same sound in Greek with the target letter. Correct responses were also: foreign words used in Greek (e.g., “snowboard”); idiomatic words (e.g., “χαψί” known as “ψάρι” (fish) in common Greek); expressions of two words functioning as an adverb [e.g., “σίμα-σίμα” (side-by-side)]; two words produced together functioning either as a noun [e.g., “χιoνoδρoμικó κέντρo” (ski center)], or as a preposition which complements the meaning of verbs, adjectives, or nouns [e.g., “σ𝜀 ξέρω” (I know you)]; two words connected with an apostrophe in written language produced as such [e.g., “άσ’τo” originating from “άσ𝜀 τo” (let it be)]; and words beginning with the target letter in written language, but this letter is part of a digraph representing a different sound in oral language [e.g., “αίνιγμα” is pronounced “enigma”]. Aside from those items repeated exactly as before, all regular inflections (i.e., different forms of verbs, nouns, adjectives, and pronouns) were counted as correct responses. The rationale for this was that children were asked to try to avoid producing the same word, but they were not instructed to avoid different forms of the same word. Wrongly articulated responses were also counted as correct responses, since there was sufficiently unambiguous evidence that a correct word has been retrieved (e.g., areoplano). There were three types of incorrect responses, each scoring zero points: repetitions; intrusions [i.e., real but irrelevant words to the target category, e.g., “ναύτης” (naftis) in the category of letter “alpha”]; and unintelligible responses (i.e., made-up words or words which could not be transcribed].

The number of phonological clusters was computed for each phonological category, where a phonological cluster was considered to be two or more successive responses that could be classified into the following types of cluster:

• Words that shared the same first syllable (e.g., σέλα-σ𝜀λήνη).
• Words that shared the same first two or more letters (e.g., σκάω-σκ𝜀πή), or sounds irrespective of spelling (e.g., χ𝜀λώνα-χαίτη).
• Words that differed only in a single vowel or consonant sound irrespective of spelling (e.g., respectively, Σίσυ-σoύσι or αδύναμoς-αδύνατoς).
• Words that shared exactly the same phonemes but spelled and pronounced slightly differently because of a different graph used for a vowel sound and a different syllable stress (e.g., χώρoς-χoρóς).
• Words that were homophones pronounced the same but spelled differently (e.g., αυτή-αυτί), identified by children when produced by using an article preceding the word which clarifies the difference in word meaning as shown in the parenthesis (e.g., αυτή-τo αυτί).
• Words that were homographs spelled the same but differed in syllable stress, and therefore pronounced differently (e.g., Σταύρoς-σταυρóς), and
• Words that were homonyms both spelled and pronounced the same, but had different meanings, identified as such by children when produced (e.g., by saying “Aγγ𝜀λική, the given name, and αγγ𝜀λική, the plant”).

In classifying words into phonological clusters, we tried to be as inclusive as possible (Mielnik et al., 2015). For example, the following sequence of words, “χαριτωμένo-χαρτί-χαρτoπ𝜀τσέτα” (cute-paper-napkin), was identified as a phonological cluster of three words, even though there is more phonological overlap between the second and the third word (χαρτ) than between the last two words with the first word (χαρ). Repeated responses, if any, were counted in computing the number and the size of clusters. The rationale is that even repeated responses might have aided children’s phonological clustering. Given that neither intrusions nor unintelligible responses could contribute to a cluster, they were not relevant for computing the number and the size of clusters. For each phonological category (i.e., “chi,” “sigma,” and “alpha”), cluster size was computed beginning with the first item in a cluster (i.e., a two-item cluster was given a size of 2). An average cluster size was then computed based on all three phonological categories. Switches were counted as the number of transitions between phonological clusters but also between non-clustered responses.

## Results

Statistical analyses were carried out using statistical package SPSS 24. The first part of the section “Results” considers the groups’ phonological fluency performance (i.e., the number of correct responses), patterns of lexical retrieval (clustering, switching, and average cluster size), and types of incorrect responses. The second part investigates the association between phonological fluency and language, literacy, and phonological skills. The third part considers the groups’ design fluency performance.

### Groups’ Performance and Group Differences on Phonological Fluency Tasks

**FIGURE 1:**
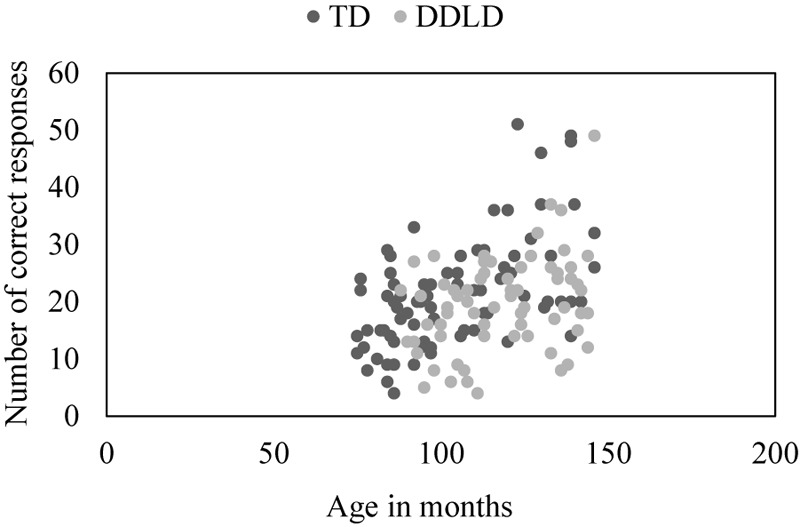
Scatterplot showing the association between the number of correct responses and age for the TD and DDLD groups.

As presented in Figure 1, Pearson correlations showed that the number of correct responses correlated strongly with age in the TD group, *r*(83) = 0.570, *p* < 0.001, and moderately in the DDLD group, *r*(66) = 0.366, *p* = 0.003. Pearson correlations also showed that nonverbal IQ performance correlated moderately with the number of correct responses in the TD group, *r*(83) = 0.398, *p* < 0.001, but did not correlate in the DDLD group, *r*(66) = 0.130, *p* = 0.297.

In order to understand whether phonological fluency performance in each group was related to the production of a greater number of clusters or to the production of more items within a cluster, partial Pearson correlations (controlling for age) were used between the number of correct responses and the number of clusters, the number of switches, and average cluster size. In the TD group, the number of correct responses correlated strongly with the number of clusters, *r*(80) = 0.743, *p* < 0.001, and the number of switches, *r*(80) = 0.828, *p* < 0.001, but not with average cluster size, *r*(80) = 0.117, *p* = 0.296. Likewise, in the DDLD group, the number of correct responses correlated with cluster number, *r*(63) = 0.724, *p* < 0.001, and the number of switches, *r*(63) = 0.788, *p* < 0.001, but again not with average cluster size, *r*(63) = 0.218, *p* = 0.081. Thus, in both groups, the production of more clusters and more switches drives word productivity, and not the production of more items within a cluster (i.e., bigger clusters).

ANCOVAs (Table 3) were carried out to assess group differences with phonological fluency variables as dependent variables, group as a fixed factor, and age in months as a covariate variable. Since the DDLD group was significantly older than the TD group, estimated marginal means and estimated standard error are presented. Analyses revealed that the TD group significantly outperformed the DDLD group with respect to the mean total number of responses, mean number of correct responses, mean number of incorrect responses, mean number of unintelligible responses, and mean number of switches. There were no group differences for repetitions and intrusions, for the mean number of clusters or for average cluster size.

**Table 3:** Groups’ performance and group differences on phonological fluency tasks.

		DDLD group		TD group				
Variables		*e. m.* mean	*e. SE*	*e. m.* mean	*e. SE*	*F*	*p*	*ηp*^2^
Total number of responses		20.15	1.01	24.04	0.90	7.816**	0.006	0.051
Number of correct responses		18.66	0.99	23.23	0.87	11.308**	0.001	0.072
Total incorrect responses		1.18	0.18	0.62	0.16	5.082*	0.026	0.034
Types of incorrect responses	Repetitions	0.28	0.07	0.19	0.06	0.854	0.357	0.006
	Intrusions	0.31	0.07	0.14	0.06	2.647	0.106	0.018
	Unintelligible	0.72	0.13	0.33	0.11	4.447*	0.037	0.030
Clusters	Number of switches	9.66	0.67	12.67	0.59	10.664**	0.001	0.068
	Number of clusters	4.39	0.32	4.99	0.28	1.800	0.182	0.012
	Average cluster size	2.60	0.10	2.60	0.09	0.000	0.992	0.000

*^∗^*p* < 0.05, ^∗∗^*p* < 0.01; *ηp*^2^, partial eta-squared, respectively, 0.01, 0.06, and 0.14 small, medium, and large effect size.*

### Relationship Between Phonological Fluency and Language, Literacy, and Phonological Skills

Table 4 presents the partial Pearson correlations (controlling for age) between the number of correct responses produced in the phonological fluency tasks and the scores for the language, literacy, and phonological tasks. Correlations are reported for the overall sample and for the DDLD and TD groups separately. In the overall sample, phonological fluency most strongly correlated with performance on WISC vocabulary and similarities subtasks. Phonological fluency also correlated with the following tasks: syntax comprehension, sentence repetition, receptive vocabulary, l’Alouette, spelling-to-dictation, phoneme deletion of CVCVCV and CCV items, nonword repetition, and rapid automatic naming. Phonological fluency did not correlate, however, with text-reading fluency (as measured by Reading Test Alpha), reading accuracy, syllable reading, nonword reading, and phoneme deletion of CVC items.

**Table 4:** Partial correlations (controlling for age) between phonological fluency (number of correct responses) and language, literacy, and phonological tasks in the overall sample and in the DDLD and TD groups.

	Overall sample		DDLD group		TD group	
Tasks	*r*	*p*	*r*	*p*	*r*	*p*
Language skills						
Verbal comprehension: WISC similarities	0.378***	<0.001	389^∗∗^	0.001	0.208	0.060
Verbal comprehension: WISC vocabulary	0.422***	<0.001	0.451***	<0.001	0.222*	0.045
Syntax comprehension: DVIQ Test	0.288***	<0.001	0.355**	0.004	0.119	0.289
Sentence repetition: DVIQ Test	0.334***	<0.001	0.390**	0.001	0.084	0.455
Receptive vocabulary: PPVT-R	0.291***	<0.001	0.153	0.224	0.246*	0.026
Literacy skills						
Text-reading fluency: L’Alouette	0.215**	0.009	0.027	0.832	0.035	0.754
Text-reading fluency: Reading Test Alpha	0.170	0.089	0.081	0.558	-0.047	0.760
Reading accuracy: Reading Test Alpha	0.112	0.267	-0.013	0.923	0.039	0.799
Syllable reading: Test of DIRD	0.105	0.488	-0.385	0.306	0.117	0.496
Nonword reading: Test of DIRD	-0.018	0.903	-0.447	0.228	0.048	0.783
Spelling ability: Spelling-to-dictation	0.363***	<0.001	0.242	0.052	0.240	0.030
Phonological skills						
Phoneme deletion of CVCVCV items: EVALEC	0.248**	0.002	0.150	0.246	0.191	0.085
Phoneme deletion of CVC items: EVALEC	0.065	0.438	0.045	0.729	0.061	0.583
Phoneme deletion of CCV items: EVALEC	0.207*	0.016	0.073	0.571	0.177	0.111
Nonword repetition: EVALEC	0.310***	<0.001	0.181	0.159	0.207	0.062
Rapid automatic naming: PhAB	-0.240**	0.003	-0.012	0.929	-0.224*	0.043

*WISC, Wechsler Intelligence Scale for Children; DVIQ Test, Diagnostic Verbal Intelligence Test; PPVT-R, Peabody Picture Vocabulary Test-Revised; Test of DIRD, Test of Detection and Investigation of Reading Difficulties; EVALEC, Evaluation de la Lecture; PhAB, Phonological Assessment Battery; ^∗^*p* < 0.05, ^∗∗^*p* < 0.01, ^∗∗∗^*p* < 0.001.*

The relationship between phonological fluency and language, literacy, and phonological tasks was investigated further. To this end, raw scores of all 11 language, literacy, and phonological tasks that correlated significantly with phonological fluency in the overall sample were converted to *z* scores. *Z*-scores were computed relative to the TD group’s mean and standard deviation for each task. The mean *z*-score was equal to 0 and *SD* equal to 1 for all tasks. *Z*-scores of all 11 tasks associated significantly with phonological fluency were entered into a PCA with oblique rotation. The PCA was carried out to explore the number of components to enter in the linear regression analyses models presented next. A component consists of measures that are correlated, with each component accounting for an amount of variance in the dataset. The amount of variance explained by a component is expressed by its eigenvalue. The dataset was suitable for the PCA: Kaiser–Meyer–Olkin Measure of Sampling Adequacy value was 0.885, meeting Kaiser’s (1974) criterion for this value, Bartlett’s Test of Sphericity value was significant (*p* < 0.001; Bartlett, 1954), and most of the intercorrelations observed among all 11 measures of interest had a value of 0.30 and above.

The PCA revealed that five language tasks (WISC similarities, WISC vocabulary, syntax comprehension, sentence repetition, and receptive vocabulary), two literacy tasks (l’Alouette and spelling-to-dictation), and four phonological tasks (phoneme deletion of CVCVCV items, phoneme deletion of CCV items, nonword repetition, and rapid automatic naming) loaded onto component 1, and that sentence repetition, phoneme deletion of CVCVCV items, phoneme deletion of CCV items, and nonword repetition additionally loaded onto component 2. Table 5 presents each task’s contribution to components 1 and 2, which is expressed by its loading value. WISC vocabulary, spelling-to-dictation, and l’Alouette had the highest loadings onto the first component, while phoneme deletion of CCV items and sentence repetition had the lowest loadings onto this component.

**Table 5:** The loadings onto components 1 and 2 for each task generated by the PCA (with oblique rotation) in the overall sample.

Tasks	Component 1	Component 2
WISC vocabulary	0.87	-0.17
Spelling-to-dictation	0.85	-0.19
L’Alouette	0.82	-0.26
WISC similarities	0.80	-0.23
Receptive vocabulary	0.79	-0.25
Rapid automatic naming	-0.73	0.18
Phoneme deletion of CVCVCV items	0.68	0.48
Nonword repetition	0.66	0.51
Syntax comprehension	0.62	-0.17
Phoneme deletion of CCV items	0.60	0.34
Sentence repetition	0.56	0.52

Components 1 and 2 had an eigenvalue larger than 1, meeting Kaiser’s (1974) criterion. The first component had, however, by far the largest eigenvalue of all 11 components generated by the PCA (5.9). The second component had an eigenvalue of 1.2 and accounted for 10% of the variance in all measures, while the remaining components had an eigenvalue lower than 1. Even though components 1 and 2 had an eigenvalue larger than 1, a one-factor solution was selected. This selection was based on the scree plot generated by the PCA illustrating a clear split between component 1 and the remaining components. The PCA was therefore repeated, and a one-factor solution was selected. This analysis revealed that component 1 had an eigenvalue of 5.9 and explained 54.24% of the variance in all 11 measures. The mean (*SD*) for component 1 was 0.00 (6.25) for the TD group and -3.54 (5.47) for the DDLD group.

Next, linear regression analysis was carried out in the overall sample with phonological fluency performance as the dependent variable, and age and component 1 as the predictors. Age was entered in the first block, and component 1 in the second block. Both age and component 1 were significant predictors; age: *Beta* = 0.447, *t* = 6.003, *p* < 0.001; component 1: *Beta* = 0.470, *t* = 5.882, *p* < 0.001, and the model was significant, *F*_(2,143)_ = 39.524, *p* < 0.001, accounting for 35.6% of the variance in phonological fluency performance. Component 1 accounted for 15.6% of the variance in phonological fluency performance. The results demonstrate that children’s language, literacy, and phonological skills significantly predict phonological fluency performance after controlling for age. Linear regression analyses by subgroup revealed a similar pattern of results. Component 1 significantly predicted phonological fluency performance in the DDLD group, accounting for 14.7% of the variance in phonological fluency performance, *F*_(2,60)_ = 12.539, *p* < 0.001; age: *Beta* = 0.384, *t* = 3.251, *p* = 0.002; component 1: *Beta* = 0.419, *t* = 3.538, *p* = 0.001. In the TD group, component 1 accounted for a smaller amount of variance in phonological fluency performance though, namely 5.7%, *F*_(2,80)_ = 24.809, *p* < 0.001; age: *Beta* = 0.570, *t* = 6.250, *p* < 0.001; component 1: *Beta* = 0.492, *t* = 2.729, *p* = 0.008.

### Groups’ Performance and Group Differences on the Design Fluency Task

Table 6 presents estimated marginal means and estimated standard error for the total number of designs, number of unique designs, and number of incorrect designs (i.e., the total number of incorrect and repeated designs). ANCOVAs were carried out to assess group differences with design fluency variables as dependent variables, group as a fixed factor, and age in months as a covariate variable. Analyses revealed that there were no group differences for the mean total number of designs, mean number of unique designs, and mean number of incorrect designs. The results demonstrate that children with DDLD do not have difficulties with design fluency, performing age-appropriately. In the overall sample, a partial (controlling for age) correlation revealed that the number of correct responses produced in phonological fluency tasks was weakly correlated with the number of unique designs generated in the design fluency task, *r*(146) = 0.268, *p* = 0.001. Therefore, in order to assess the specificity of the phonological fluency deficit in children with DDLD, an ANCOVA was carried out, with the number of correct responses in phonological fluency tasks as a dependent variable, group as a fixed factor, and age in months and the number of unique designs generated in the design fluency task as covariate variables. ANCOVA revealed that there was still a group difference for the mean number of correct responses produced in phonological fluency tasks, *F*_(1,145)_ = 9.687, *p* = 0.002, *ηp*^2^ = 0.063. The result demonstrates that after the effects of age and design fluency performance were controlled, children with DDLD still show lexical retrieval difficulties in phonological fluency tasks, arguing for the specificity of the phonological fluency deficit in children with DDLD.

**Table 6:** Groups’ performance and group differences on the design fluency task.

	DDLD group		TD group				
Variables	*e. m.* mean	*e. SE*	*e. m.* mean	*e. SE*	*F*	*p*	*ηp*^2^
Total number of designs	24.55	0.95	25.09	0.84	0.133	0.716	0.001
Number of unique designs	20.25	0.72	21.53	0.64	1.655	0.200	0.011
Number of incorrect designs	4.30	0.51	3.49	0.45	1.277	0.260	0.009

*ηp^*2*^, partial eta squared, respectively, 0.01, 0.06, and 0.14 small, medium, and large effect size.*

## Discussion

The aims of this study were to investigate phonological fluency in Greek children with DDLD aged 7–12 years by comparing DDLD and TD children’s clustering patterns, toward teasing apart two theoretical hypotheses accounting for the locus of the phonological deficit in dyslexia and DLD. Children with dyslexia and/or DLD were combined in one group, the DDLD group, based on evidence from a PCA conducted within the language and literacy skills of the DDLD group which revealed that DDLD children’s language and literacy skills loaded onto a single component. This finding was interpreted as evidence that dividing the DDLD group into separate subgroups was not appropriate in the current study. We further investigated how phonological fluency performance is related to children’s language, literacy, and phonological skills. We also tested the specificity of the phonological fluency deficit in children with DDLD by using a design fluency task not requiring phonological representations and phonological processing skills.

Three phonological categories, namely “chi,” “sigma,” and “alpha” of the Greek alphabet, were used, and a composite phonological fluency score was computed. In both groups, phonological fluency performance was driven by the number of clusters and the number of times children switched between clustered- and/or non-clustered responses, but not by the size of clusters. Children with DDLD produced fewer correct responses than TD children after controlling for age. This finding is consistent with previous studies in children with dyslexia speaking languages other than Greek (e.g., in English: Brosnan et al., 2002; in French: Plaza et al., 2002; in German: Landerl et al., 2009; in Italian: Varvara et al., 2014; in Portuguese: Moura et al., 2015), and in English-speaking children with DLD (Weckerly et al., 2001; Henry et al., 2012, 2015). Although the TD group produced significantly fewer incorrect responses than the DDLD group, both groups produced low numbers of incorrect responses (repetitions, intrusions, and unintelligible responses).

Further, children with DDLD produced significantly fewer switches than TD children, but the two groups did not differ significantly on the number of clusters or on average cluster size. The findings in turn suggest that children with DDLD produced fewer correct responses compared to TD children because they did not switch as many times, and not because they did not identify as many clusters or because their clusters were smaller. Previous evidence reported no significant difference in cluster size between adolescents with dyslexia aged 16–18 years and controls (Mielnik et al., 2015), and also between English-speaking children with DLD and age-matched TD children (Weckerly et al., 2001). This is the first study which considered phonological cluster size in Greek children with dyslexia and/or DLD, and found a similar average cluster size between children with and without DDLD, replicating those previous findings in languages other than Greek just presented above. This is despite the DDLD group having poorer phonological skills as measured with phonological awareness, phonological short-term memory, and rapid automatic naming tasks.

In relation to both phonological hypotheses considered, the finding that children with DDLD produced significantly fewer words than TD children implies that in an explicit task in which phonological representations and phonological processing skills are involved, phonological representations in children with DDLD are less accessible compared to TD children. The finding, however, that children with DDLD produced a similar size of clusters relative to TD children is interpreted as indicating that children with DDLD performed age-appropriately in an implicit task in which phonological processing is minimized given that phonological clustering is considered to measure children’s ability to access phonological representations implicitly. Together the two findings thereby suggest that, as proposed by the Deficient Phonological Access Hypothesis, children with DDLD show deficient explicit access to phonological representations affecting lexical retrieval processes, but intact implicit access to them.

Children’s language, literacy, and phonological skills were associated with phonological fluency scores. The PCA revealed one component defined by tasks of verbal comprehension, syntax comprehension, sentence repetition, receptive vocabulary, text-reading fluency, spelling, phoneme deletion, nonword repetition, and rapid automatic naming. This component was a significant predictor of phonological fluency performance after controlling for age, explaining 15.6, 14.7, and 5.7% of the variance in phonological fluency performance in the overall sample, in the DDLD group, and in the TD group, respectively. This finding suggests that children with DDLD achieved lower phonological fluency scores than TD children partly because they had poorer language, literacy, and phonological skills.

Nonverbal fluency performance was compared in the two groups using a design fluency task which measures visuospatial executive skills. The two groups generated a similar number of unique designs, suggesting that children with DDLD show a fluency deficit specific to the phonological aspects of language, and not general speed processing difficulties which might have resulted in lower phonological fluency performance. However, children with DDLD showed poorer phonological fluency performance even after controlling for design fluency performance in the analysis. This finding supports the specificity of the phonological fluency deficit, as suggested by the two phonological hypotheses considered. In addition, another measure thought to reflect executive skills is switching (e.g., Troyer, 2000; Bertola et al., 2014), and children with DDLD switched significantly less often than TD children. This finding might suggest that phonological fluency tasks loaded executive skills in children with DDLD, and concomitantly, that, if this was the case, poorer phonological fluency performance was limited by executive skills involved in the phonological fluency tasks. One explanation might be that given that semantic search is the default search strategy that people use to scan the mental lexicon (Vonberg et al., 2014), producing responses in phonological categories requires executive skills to a greater extent than other types of fluency tasks, such as semantic fluency tasks (e.g., Kavé et al., 2008; Shao et al., 2014; Smith-Spark et al., 2017).

A strength of the study is its large overall sample, which covers a wide range of verbal fluency, design fluency, language, literacy, and phonological scores, and the large numbers of TD children and children with DDLD separately. As West et al. (2018) argue, this allowed the investigation of the associations between measures without over-estimating the size of any association and avoided results of low statistical power which yield many false positive results. A limitation is that the concept of phonological access is underspecified in the literature. As Mirman and Britt (2014) argue in the context of semantic access deficits in adults, it is not clear precisely what researchers mean when they refer to “access,” nor what the nature of the “access deficit” is. Further investigation, using different research methods, is needed to shed light on the origin of phonological access deficits in dyslexia and DLD. To this end, Boets et al. (2013) reported that in adults with dyslexia less coordination was found between brain regions in the bilateral auditory cortex that process basic phonemes and Broca’s region, a region in the brain’s frontal lobe known to be involved in higher-level language processing. The researchers interpreted this evidence as suggesting that deficient access to phonological representations originates from the above-mentioned disconnection between cortical regions and Broca’s region in adults with dyslexia. It remains to be investigated though whether this finding can be replicated in a sample of children with dyslexia and DLD.

To conclude, the objective of this study was to investigate the locus of the phonological deficit in a sample of Greek children with DDLD by investigating the structure of phonological fluency. To this end, two theoretical hypotheses were considered which attempt to explain where the phonological deficit in dyslexia and DLD lies, namely in children’s phonological representations or in children’s ability to explicitly access (intact) phonological representations. The children with DDLD retrieved fewer correct items in phonological fluency tasks than did TD children, and they also switched less often between clustered and/or non-clustered responses. However, a similarly sized average cluster, considered to be an implicit phonological measure of the quality of phonological representations, suggested that in children with DDLD phonological representations were as robust and distinct as those of TD children. This is consistent with the Deficient Phonological Access Hypothesis. The finding that children with DDLD showed poorer phonological fluency performance relative to TD children even after controlling for the effect of design fluency performance supported the specificity of the phonological fluency deficit in children with DDLD on the basis that only phonological processing difficulties, and not general processing speed difficulties underlie poorer phonological fluency performance in children with DDLD. This finding is consistent with the two prominent phonological hypotheses considered in the current study. Children’s language, literacy, and phonological skills predicted phonological fluency performance, suggesting that poorer phonological fluency performance in children with DDLD is partly attributed to their inferior language, literacy, and phonological skills. Further investigation is needed to shed light on the underlying cause(s) of deficient explicit access to phonological representations in children with dyslexia and DLD.

## Author Contributions

MM and CM designed the study, contributed to manuscript revision, and read and approved the submitted version. MM collected and analyzed the data, and wrote the first draft of the manuscript. CM made a significant contribution to interpreting the data and structuring the manuscript.

## Conflict of Interest Statement

The authors declare that the research was conducted in the absence of any commercial or financial relationships that could be construed as a potential conflict of interest.

## Footnotes

1 The term “DLD” has been proposed as a replacement for “Specific Language Impairment” (Bishop et al., 2017).

## Abbreviations

DDLD: Dyslexia and/or Developmental Language Disorder
DLD: Developmental Language Disorder
PCA: principal component analysis
